# Neurofibrillary tangle-bearing neurons have reduced risk of cell death in mice with Alzheimer’s pathology

**DOI:** 10.1016/j.celrep.2024.114574

**Published:** 2024-08-02

**Authors:** Theodore J. Zwang, Eric del Sastre, Nina Wolf, Nancy Ruiz-Uribe, Benjamin Woost, Zachary Hoglund, Zhanyun Fan, Joshua Bailey, Lois Nfor, Luc Buée, K. Peter R. Nilsson, Bradley T. Hyman, Rachel E. Bennett

**Affiliations:** 1 Department of Neurology, Massachusetts General Hospital, Charlestown, MA, USA; 2 Harvard Medical School, Boston, MA, USA; 3 University Lille, Inserm, CHU Lille, LilNCog—Lille Neuroscience & Cognition, 59000 Lille, France; 4 Division of Chemistry, Department of Physics, Chemistry and Biology, Linköping University, 581 83 Linköping, Sweden; 5 These authors contributed equally; 6 Lead contact

## Abstract

A prevailing hypothesis is that neurofibrillary tangles play a causal role in driving cognitive decline in Alzheimer’s disease (AD) because tangles correlate anatomically with areas that undergo neuronal loss. We used two-photon longitudinal imaging to directly test this hypothesis and observed the fate of individual neurons in two mouse models. At any time point, neurons without tangles died at >3 times the rate as neurons with tangles. Additionally, prior to dying, they became >20% more distant from neighboring neurons across imaging sessions. Similar microstructural changes were evident in a population of non-tangle-bearing neurons in Alzheimer’s donor tissues. Together, these data suggest that nonfibrillar tau puts neurons at high risk of death, and surprisingly, the presence of a tangle reduces this risk. Moreover, cortical microstructure changes appear to be a better predictor of imminent cell death than tangle status is and a promising tool for identifying dying neurons in Alzheimer’s.

## In brief

Zwang et al. used two-photon microscopy to track neurofibrillary tangle (NFT) formation and neuron loss in model mice. They observed that neurons with NFTs died less frequently than neurons without NFTs. Dying neurons could also be identified by local cortical microstructure changes, providing a method to identify susceptible populations.

## Graphical Abstract

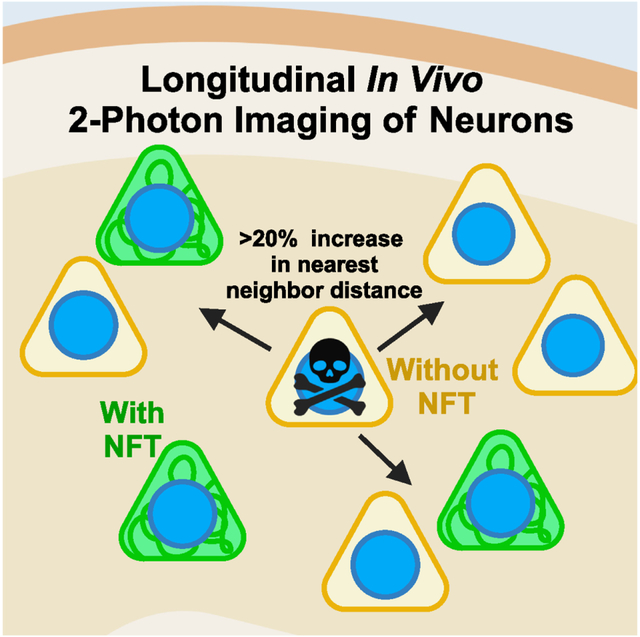

## INTRODUCTION

The presence of neurofibrillary tangles (NFTs), and other aggregates of neurodegeneration-associated proteins, has long been assumed to cause neurotoxicity and ultimately neuronal death. NFTs form in a hierarchical pattern across specific brain regions and lamina as Alzheimer’s disease (AD) progresses, and those are frequently areas that undergo marked neuronal loss.^1–3^ Therefore, the idea that tangles are neurotoxic remains a central dogma in AD research.

However, quantitative studies show that neuronal loss outstrips tangle formation by nearly 10-fold in both human AD and mouse models.^4,5^ Two plausible possibilities for the quantitative disparity between NFT number and loss of neurons are that (1) tangles kill neurons and then ultimately disappear, or (2) neurons without a tangle die. Previous work has shown that NFT-containing neurons are under substantial stress, but the presence of a tangle, even a tangle in a neuron containing caspase activation, was not associated with acute toxicity within 5 days of observation. However, these experiments did not directly observe neurons dying, with or without tangles, and lacked the ability and time resolution necessary to resolve whether tangles are a protective factor or associated with slow or delayed toxic effects on neurons. To definitively test the idea that NFTs are a precursor to neuronal death, we used longitudinal *in vivo* two-photon microscopy to directly observe the relationship between tangle presence and survival of individual neurons in the rTg4510 and ThyTau22 mouse models. These mice overexpress human tau containing the P301L or G272V/P301S mutations, respectively, and develop tangles and neuron loss with age.^6–8^ Over multiple weeks, hundreds of non-tangle-bearing and tangle-bearing neurons were observed simultaneously in live mice, including numerous cells that developed tangles during the imaging period. Rare neurons died during the imaging interval. Contrary to expectations, these data show that tangle-bearing neurons are significantly more likely to survive than non-tangle-bearing neurons in mice with human tau expression. Moreover, we were able to detect a phenotype in mouse tissues that predicted a high risk of impending neuronal death. This phenotype—a change in the local microstructure of the cortex—was also observed primarily in non-tangle-bearing neurons in 3D postmortem studies of human AD patients, consistent with the idea that non-tangle-bearing neurons are at higher risk of dying than tangle-bearing neurons in both mouse models and human disease.

## RESULTS

### Tracking neurons *in vivo*

**Figure 1.**
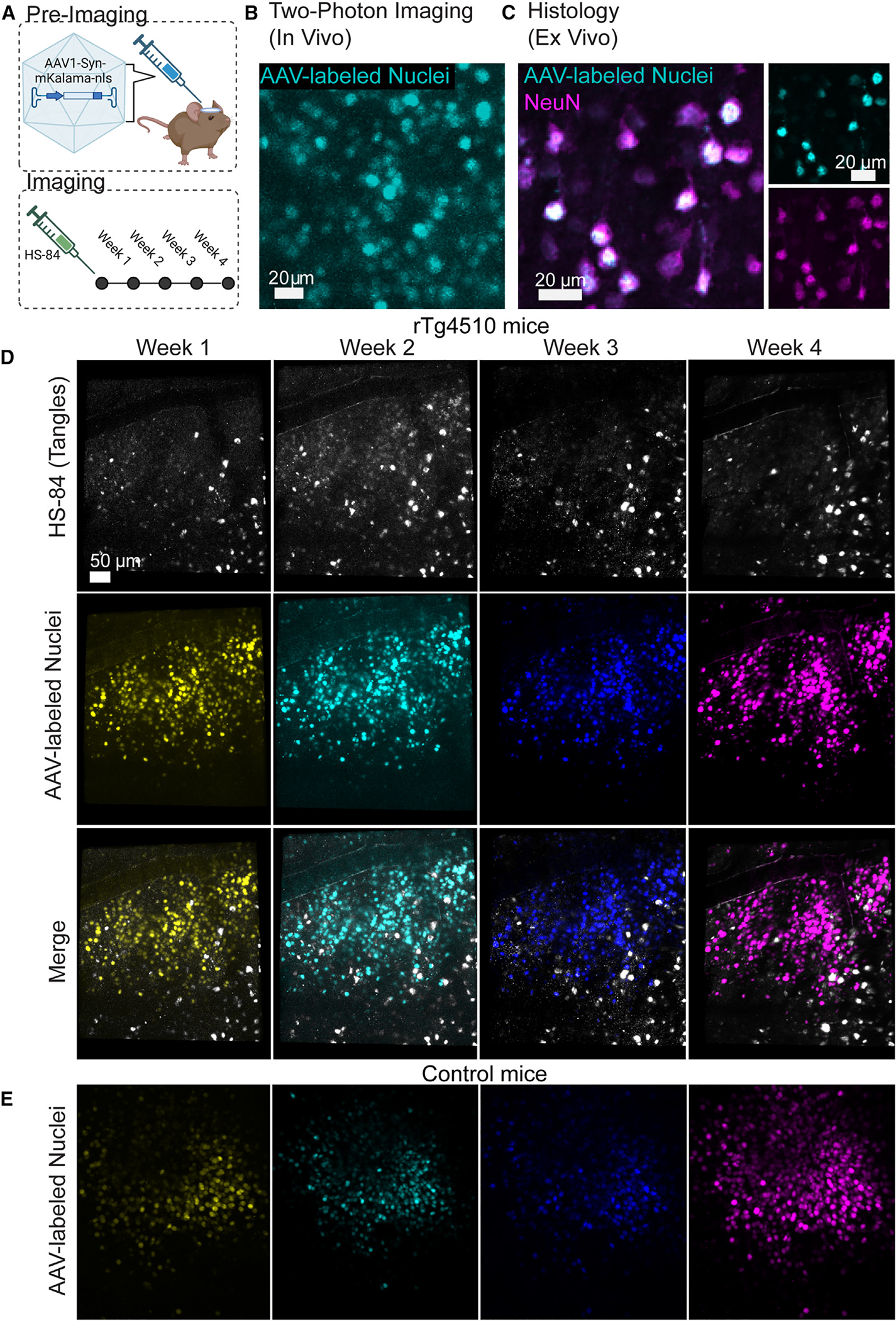
Experimental design and analysis methods for tracking individual neurons and the formation of tangles over 4 weeks (A) Overview of timeline and methods used in these experiments. (B) AAV labeling of neuronal nuclei was visualized *in vivo* by two-photon microscopy. (C) Immunofluorescence confirms that the nuclear labeling strategy used labels NeuN^+^ neurons. (D and E) Example of HS-84 tangles and labeled nuclei imaged across 4 weeks (D) in a rTg4510 mouse and AAV-labeled nuclei (E) in a control mouse. Images are color-coded by week.

**Figure 2.**
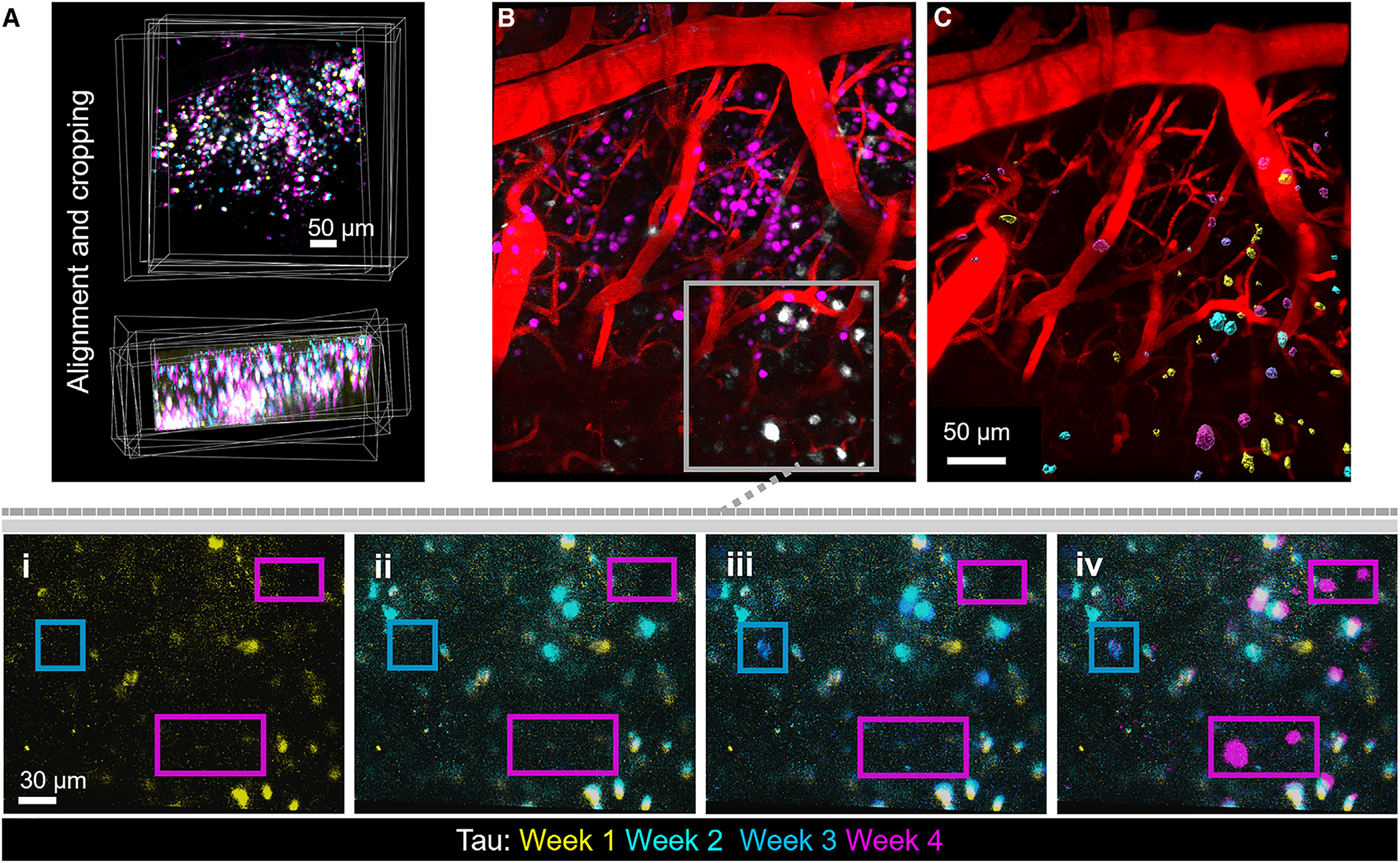
Identifying new neurofibrillary tangle formation in live mice (A) Image volumes were aligned and cropped to enable quantitative assessment of tangle formation and neuron death across weeks. (B) New tangles were identified based on the appearance of HS-84-labeled objects that were not present the previous week. Image shows labeled neuronal nuclei in magenta, intravenous dextran-labeled blood vessels in red, and tangles in white. Higher magnification images of the boxed area from each week are shown in panels (i–iv). The blue box shows a new tangle appearing on week 3, and the magenta box shows new tangles appearing on week 4. (C) All new tangles were registered and pseudo-colored based on their week of appearance: week 1 (yellow), week 2 (cyan), week 3 (blue), and week 4 (magenta).

To study tangles *in vivo*, we prepared 4-month-old rTg4510 or control mice for longitudinal imaging of neurons and NFTs (Figure 1A). The rTg4510 mouse line develops robust NFT pathology and cortical atrophy throughout the somatosensory cortex, an area readily imaged with two-photon microscopy.^6,7^ Control mice carry the tetracycline responsive element transgene but not the tetracycline-controlled transactivator protein (tTA) allele that drives tau expression in forebrain neurons. Neuronal nuclei were then labeled using intracortical injections of an adeno-associated virus (AAV) carrying an expression cassette for a cyan fluorescent protein (mKalama1 variant) with a nuclear localization signal (NLS) driven by the synapsin promoter. After initial testing *in vivo* in a control mouse (Figure 1B), the specificity of nuclear labeling in neurons was confirmed ex vivo using immunofluorescence (Figure 1C). 3 weeks after AAV injection and installation of cranial windows for longitudinal two-photon imaging, the luminescent conjugated oligothiophene dye HS-84 was introduced via intravenous injection. This highly fluorescent two-photon compatible dye has been shown to bind tau NFTs with high signal to noise *in vivo*.^9–11^ Importantly, HS-84 can persistently label new and old NFTs for multiple weeks after a single injection because it is not rapidly metabolized and remains in circulation post injection.^10^ In this work, we observed the ability for HS-84 to stably label old tangles and to label newly formed tangles for 4 weeks without requiring re-injection. An example of a typical tangle-bearing neuron is shown in Figure S1. Mice were imaged every week for 4 consecutive weeks (Figures 1D and 1E). After aligning overlapping regions shared across weekly imaging sessions, we quantified the appearance of NFTs and death of neurons in these regions of interest (Figures 2A–2C). With this method, we were able to track 1,721 total neurons including 235 neurons with tangles and 1,486 neurons without tangles in rTg4510 mice (*n* = 6 mice) and 1,589 non-tangle-bearing neurons in control mice (*n* = 4 mice). Across all rTg4510 mice, we initially counted 128 tangles and watched 107 tangles appear, with an average of 10.6 ± 4 forming each week. This confirms earlier observations that tangles can form rapidly, within days.^12^

### Impact of neurofibrillary tangles on neuron loss

**Figure 3.**
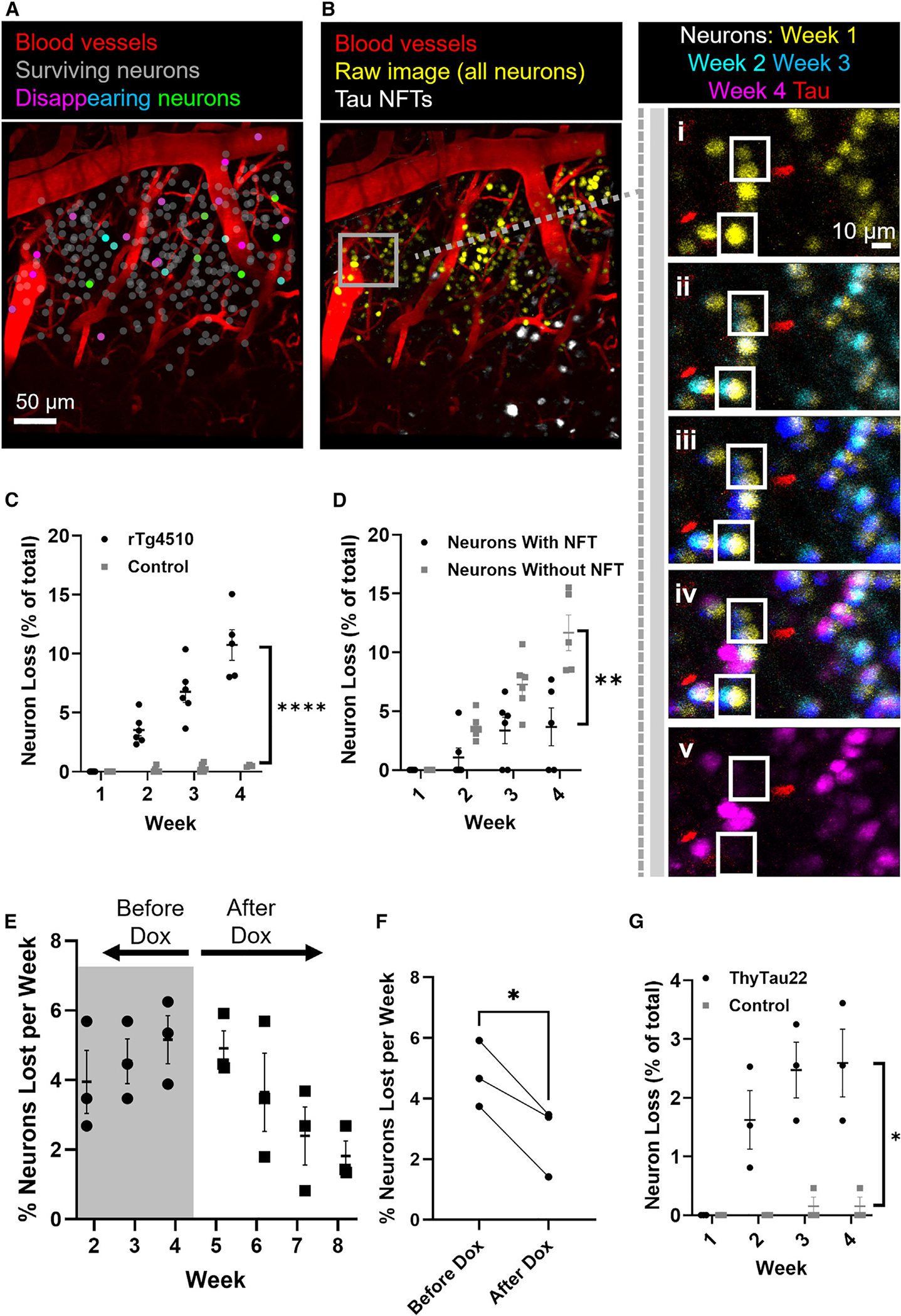
Neurofibrillary tangles protect neurons against cell death (A) All of the labeled neurons were registered to the first week’s data. Neurons that persisted across all weeks are labeled in white, and neurons that disappeared are color-coded based on the week of disappearance: week 2 (green), week 3 (teal), week 4 (magenta). (B) Image of all neurons (yellow) and tangles (white) from week 1. Boxed area is highlighted with higher magnification images of each week (i–iv) showing non-tangle-bearing neurons that disappear between weeks 3 and 4. (v) shows week 4 only, confirming the absence of these neurons. (C) Total neuron loss in rTg4510 mice (black; cohorts 1 and 2, *n* = 6) and in littermate control mice (gray, cohort 1, *n* = 4). Mixed effects analysis is of effect of week (*p* < 0.0001), genotype (*p* < 0.0001), and week x genotype (*p* < 0.0001). (D) Comparison of neuron loss in rTg4510 neurons with tangles (gray) and rTg4510 neurons without tangles (black). Plots represent measures of all neurons from cohorts 1 and 2 (rTg4510 mice; *n* = 6 individuals). Mixed effects analysis effect of week (*p* < 0.0001), tangle status (*p* = 0.0037), week x tangle status (*p* = 0.0002). (E) Neuron loss for *n* = 3 independent rTg4510 mice (cohort 2) before and after the administration of doxycycline (Dox). (F) Statistical comparison of neurons lost per week in rTg4510 mice (*n* = 3, cohort 2), using a paired t test to compare the average percent of neurons lost across all weeks before and all weeks after doxycycline (*p* = 0.016). (G) Total neuron loss in 15-month-old ThyTau22 mice (black, *n* = 3) and in littermate control mice (gray, *n* = 4). Mixed effects analysis is of effect of week (*p* = 0.0065), genotype (*p* = 0.015), week x genotype (*p* = 0.0002). Error bars are means ± SE. *p < 0.05, **p < 0.01, and ****p < 0.0001.

We then quantified the death of neurons based on the disappearance of mKalama1-positive fluorescent nuclei and classified them as tangle-bearing or non-tangle-bearing based on the presence of HS-84 labeling (Figures 3A and 3B). In control mice (*n* = 4), only non-tangle-bearing neurons were present, and 8 out of 1,589 (0.5%) were not apparent on re-imaging at any point over subsequent weeks of imaging. In rTg4510 mice (*n* = 6), a total of 178 out of 1,721 (10.3%) neurons disappeared. Neuron loss across weeks was dramatically increased in rTg4510 mice, as expected (Figure 3C). Importantly, when we examined tangle-bearing and non-tangle-bearing neurons using a mixed effects model we observed a statistically significant effect of tangle status on the rate of neuron death; substantially fewer tangle-bearing neurons die each week (*p* = 0.0037, Figure 3D). Overall, we observe that 6 of 235 (2.6%) tangle-bearing neurons died, whereas 172 of 1,486 (11.6%) non-tangle-bearing neurons died, resulting in a highly statistically significant difference (chi-squared test = 17.8, degrees of freedom = 1, *p* < 0.0001; Table S1). Additionally, 3.62% ± 0.35% of neurons were lost each week in rTg4510 mice, which is in line with previous reports that used stereology and estimated 4% ± 1% of neurons lost per week in the cortex of this mouse model.^5^ These data are also summarized by the hazard ratio comparing the death rate of neurons with and without tangles, showing that neurons without tangles were more than three times as likely to die in any single week (hazard ratio = 3.59 ± 1.05) than those with tangles in the same rTg4510 mice and 19.91 ± 3.43 times more likely to die than those without tangles in control mice. This suggests that developing a tangle is a phenotype associated with reduction in relative risk of dying from tau overexpression.

To further explore this possibility, we turned off tau gene expression via the doxycycline-repressible promoter in a cohort of rTg4510 mice and hypothesized that if soluble nonfibrillar tau species are responsible, then repressing tau expression should result in lessened cell loss over time. In this group, after an initial observation period of 4 weeks, mice were re-injected with HS-84, fed a diet containing doxycycline, and imaged weekly for an additional 4 weeks. This experiment shows that the rate of neuron loss from week to week was 4.8% (±1.4%) prior to the start of doxycycline treatment and then improved to 2.4% (±0.9%) after 4 weeks of tau suppression (Figure 3E); i.e., the rate of neuronal death was cut in half despite the fact that the estimated half-life of tau is on the order of weeks, suggesting that tau levels remained high for at least part of the doxycycline treatment period. As expected, doxycycline treatment for only 1 month did not affect overall cortical atrophy or tangle pathology but did result in a reduction in soluble tau levels by 62% (±12%) as measured by western blot (Figure S2). Pairwise comparisons between the percent of neurons lost on average during the weeks prior to doxycycline treatment versus that final week of treatment show there is a significant reduction in neuron loss (*p* = 0.02; Figure 3F). These results provide direct evidence that NFTs, per se, are not toxic to neurons.

We then compared neuron loss in a second, less aggressive transgenic tau model, aged Thytau22 mice. In this line, we observed 845 total neurons in transgenic mice over 4 weeks and 24 of them disappeared (2.8%; Figure 3G). When we compared ThyTau22 and control mice using a mixed effects model, we observed that ThyTau22 mice have significantly more dying neurons (*p* = 0.0156), a significant increase in the number of dying neurons from week to week in Thytau22 mice (*p* = 0.0065), and significant interaction between week and genotype (*p* = 0.0002). No neurons with tangles were observed, which is in line with the limited cortical tangle formation at this age in this strain (Figures S2A–S2C).^8^ By comparison, 860 neurons were observed in wild-type control littermates, of which only 1 disappeared (0.1%) over the course of the experiment. This further supports the idea that NFTs do not directly contribute to the loss of neurons and indicates that soluble, rather that fibrillar tau, may be more related to neuron death.

### Alterations in local microstructure preceding neuron loss

**Figure 4.**
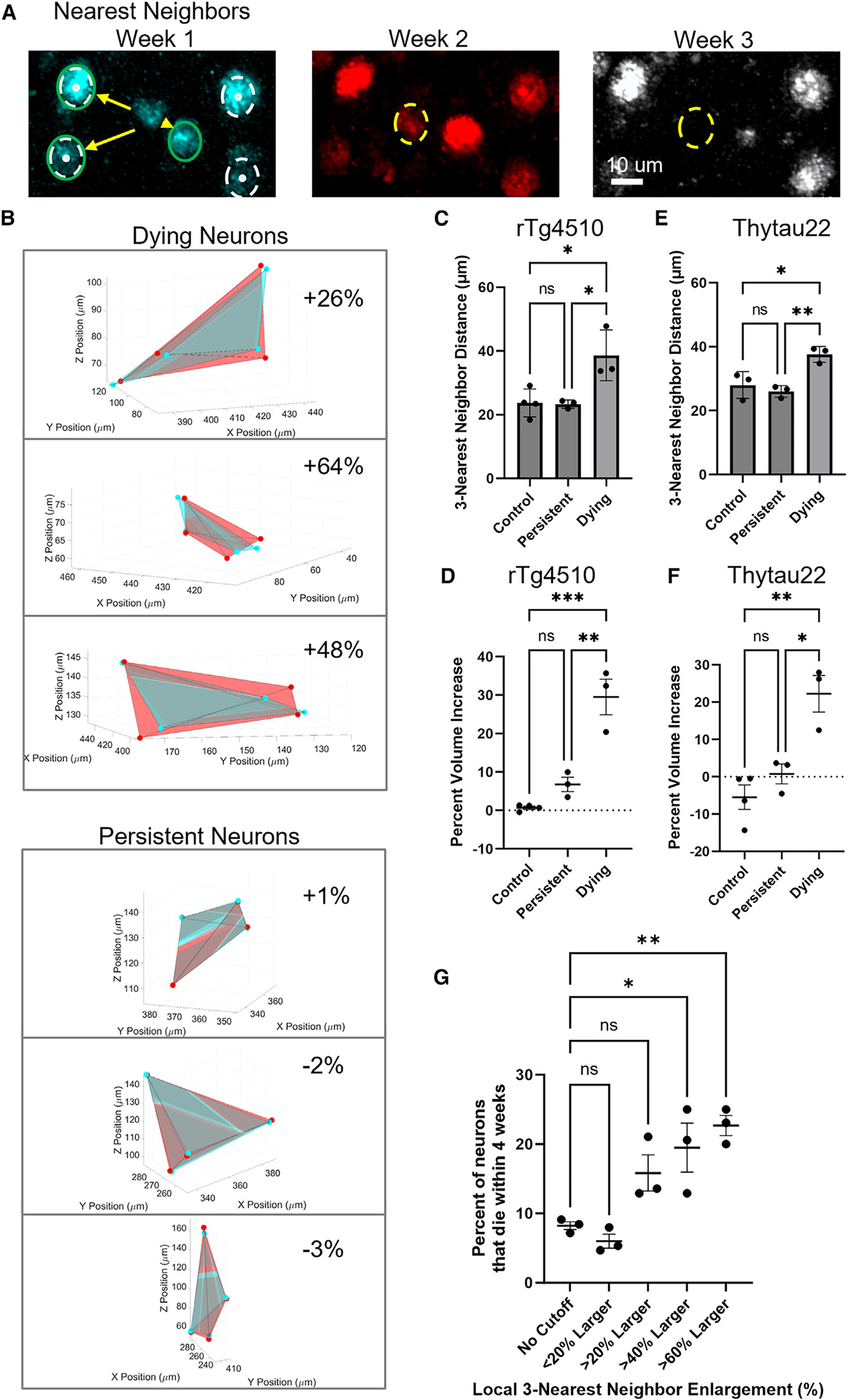
Dying neurons show alterations in local microstructure that precede their demise (A) Disappearing neurons (yellow dashed circle) were identified by their loss of fluorescence across weeks. The distance from the disappearing neurons to its 3-nearest-neighboring neurons (green circle, yellow arrows) was determined regardless of the fate of those nearby neurons. To measure changes over time, we found the center points of 4 nearby persistent neurons (white dashed circle). (B) The center points of nearby persistent neurons were used as vertices to create shapes whose volume change could be measured over time. The cyan polyhedron was made from the center point of nearby neurons from the first week, and the red polyhedron is from the neuron’s position 1 week later. (Top) Representative examples wherein increasing distances between neurons were observed across consecutive weeks before disappearance in rTg4510 mice and (bottom) persistent neuron distances in control mice. (C and E) In rTg4510 (C; cohort 1, one-way ANOVA *p* = 0.01) and in Thytau22 (E; cohort 3, one-way ANOVA *p* = 0.007), the average distance between a disappearing neuron and its 3 nearest neighbors during the first week of observation tends to be larger than the average distance for nearby (<30 μm) neurons that are persistent or neurons in control mice. (D) Changes in the volume of the shape formed by the vertices of 4 nearby neurons show that disappearing neurons have a significant increase in the weeks prior to disappearance compared to control neurons and persistent neurons (one-way ANOVA *p* = 0.0002 and *p* = 0.0014), while persistent neurons and control neurons do not change in size over time in rTg4510 mice (cohort 1). (F) Dying neurons also changed in volume in ThyTau22 mice when compared to control neurons and persistent neurons (right; cohort 3; one-way ANOVA *p* = 0.0025 and *p* = 0.014). (G) Percent of neurons within each rTg4510 mouse that died within the 4 weeks of observation based on comparison to local nearest neighbor distances as cutoff values (one-way ANOVA corrected for multiple comparisons. From left to right, *p* = 0.9236, 0.1163, 0.0156, and 0.0029). Each point is an independent mouse. Error bars are means ± SE. Asterisks indicate Tukey’s multiple comparisons, *p < 0.05, **p < 0.01, ***p < 0.001, and n.s. = not significant.

Recent studies suggest that neurons at risk for death in tissue culture models may display morphological features that predict vulnerability.^13^ We re-examined *in vivo* images of neurons that were identified as destined to die and explored the possibility that there would be a morphological change associated with neurodegeneration. To examine local microstructure, we measured the distance between a neuron and its three nearest neighbors 1 or 2 weeks prior to loss (Figures 4A and 4B). We observed that dying neurons in rTg4510 mice are significantly farther away from neighboring neurons than persisting neurons (33.9 μm ± 1.9 μm compared to 24.0 μm ± 0.5 μm, *p* = 0.02; Figure 4C). These dying neurons are also farther away from their neighbors than neurons in control mice (22.4 μm ± 0.3 μm, *p* = 0.02).

We then tested whether this reorganization around dying neurons changes over time by identifying the 44 dying neurons from transgenic mice that had at least 2 weeks of measurements prior to their disappearance and that had 4 nearby neighbors that persisted across all weeks. Using the neighboring neurons’ center points as vertices allowed us to generate shapes and measure the volume contained within each shape (Figures 4A and 4B). In control mice, 44 randomly chosen neurons showed essentially no change with an average neighbor-volume difference across weeks of 0.7% ± 4.0% (*n* = 4 mice; Figure 4D). In rTg4510 mice, even though dying neurons started at larger distances from their neighbors, the volume in between their neighboring neurons continued to increase an average of 29.5% ± 4.6% (*p* = 0.0002 vs. control). This is roughly equivalent to a 2.2 μm ± 0.3 μm increase in distance between nearest neighbors across the 2 weeks prior to the neurons’ disappearance. In comparison, the 44 randomly chosen persistent neurons from the three rTg4510 mice showed a non-statistically significant average neighbor-volume change of 6.8% ± 1.9% (*p* = 0.0014 vs. control). Across mice, persistent neurons with an NFT showed no significant difference in neighbor-volume change across weeks, 1.5% ± 4.5%, when compared to neurons without an NFT, 7.3% ± 2.0% (*n* = 3 mice, *p* = 0.26). In rTg4510 mice, neurons that show an increasing displacement of their neighbors across 2 weeks have a 2.2 times increased risk of imminent death in the following week, when compared to neurons that do not have this phenotype (*p* = 0.0015; Table S2). Neurons whose neighbor-volume increases >20% are at extraordinary risk of dying, with 46.7% (±3.3%) dying the following week. A similar disruption of microstructure was also observed in the Thytau22 mouse line (Figures 4E and 4F). From these data, it appears that local microstructure disruption predicts the demise of neurons, on the order of ~2 weeks prior to their disappearance.

For many experiments, such as studies in postmortem brain, it is not possible to collect longitudinal images of live neurons and how they change across weeks. We wanted to determine whether successful predictions of neuron death could be made from images taken at a single time point. Rather than measuring change in nearest neighbor distances over time, we evaluated cortical microstructure in mice at a single time point, to identify neurons with substantially increased 3-nearest-neighbor distances compared to other neurons within 100 μm (Figure 4G). After identifying neurons with altered intra-distance measurements at a single time point, we used the subsequent images from following weeks to determine their fate. We found that neurons with a >60% increase in 3-nearest-neighbor distances compared to nearby neurons at an initial time point were 2.75 times more likely to die over the next 4 weeks than the general population (*p* = 0.0025), with 22.7% (±1.5%) dying over the subsequent period of observation. Thus, while less powerful than having 4-dimensional, time-resolved data, interrogation of neuronal microstructure from static images is sufficient to identify neurons at high risk of dying.

### Identifying microstructural disruptions in human inferior temporal gyrus

**Figure 5.**
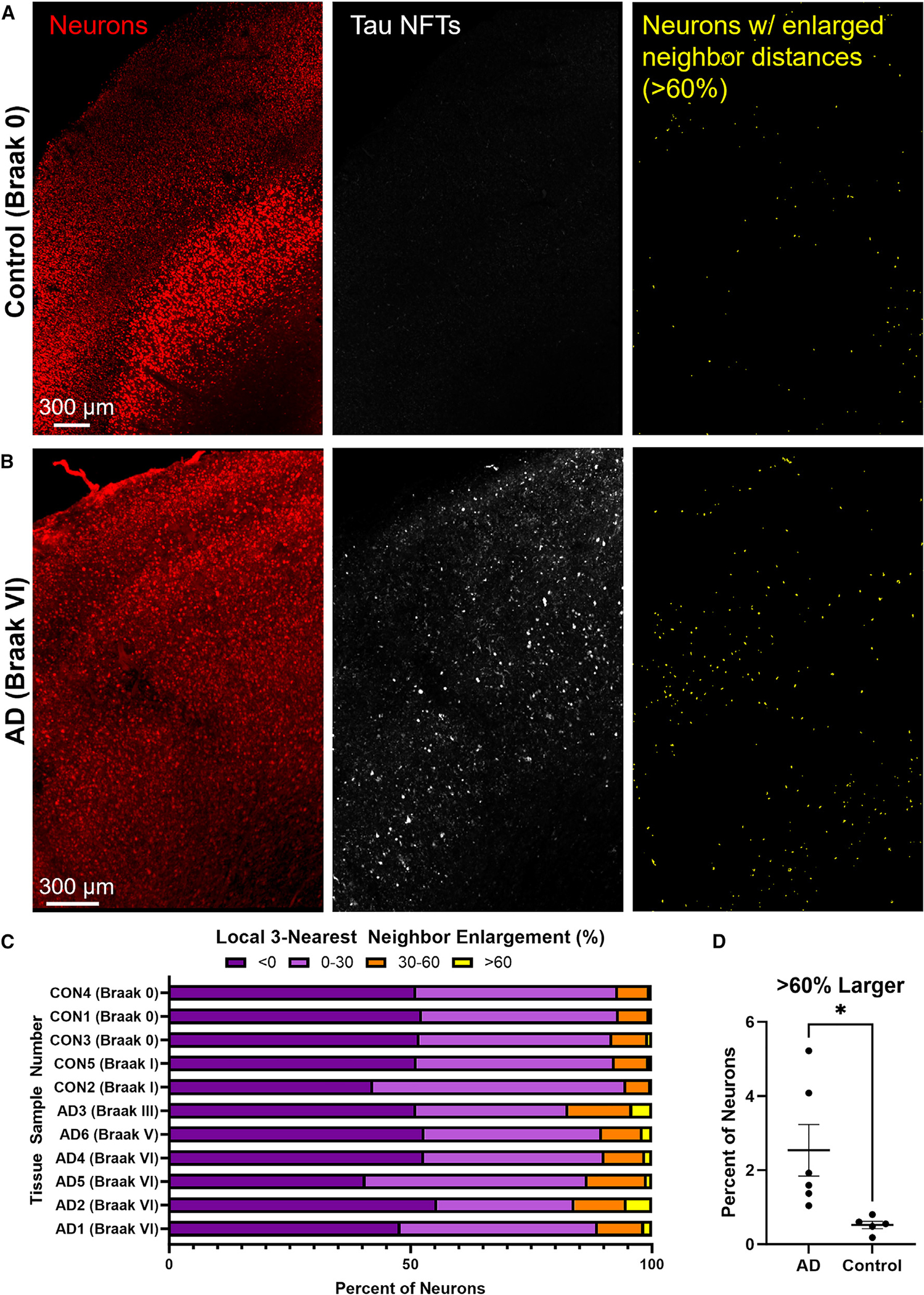
Human AD accumulates neurons with enlarged nearest neighbor distance (A and B) Representative examples of neuron segmentation and nearest neighbor distances from (A) control (*n* = 5) and (B) AD (*n* = 6) human inferior temporal gyrus tissue. Images represent Z-projection of neurons stained with HuD (red) and neurofibrillary tangles stained with AT8 (white) through 500-μm tissue slice. Neurons were isolated using Ilastik pixel and object classification, and neurons with >60% enlargement of their 3-nearest-neighbor distance compared to all other neurons within 100 mm are represented in yellow. Non-neuronal objects as determined by the object classification were removed from this visualization. (C) Bar graph showing the proportion of all neurons for each group within that tissue sample. (D) Percent of neurons with >60% enlargement from each tissue sample. Two-tailed t test shows significantly increased number of neurons in this group for AD tissue compared to control tissue (*p = 0.024). Error bars are means ± SE.

To determine whether an analogous disruption of local cortical microstructure is also present in human AD, we cleared, stained, and imaged 0.5- to 1-mm-thick sections of human brain tissue from the inferior temporal gyrus of 11 donors (Figures 5A–5D). This includes 6 AD donors with frequent tangles (Braak stage III/V/VI) and the 5 control donors without tangles in the region examined (Braak stage 0/I). We used pixel classifier and object classifier methodology to identify and segment individual neurons. Each image contained between 18,485 and 162,383 segmented neurons for a total 268,849 neurons identified in AD tissue and 425,540 neurons identified in control tissue.

We then identified neurons with enlarged nearest neighbor distances by calculating the 3-nearest-neighbor distance for each neuron and dividing by the average 3-nearest-neighbor distance for all nearby neurons (<100 μm). These data show that neurons had characteristics similar to what we had observed in mice: the local microstructural environment showed enlarged distances between neurons for a very small subset of neurons in AD, and far fewer of these neurons were observed in control tissue. This analysis did not preferentially identify neurons at the edge of tissue or around defects in the tissue, and neurons were distributed throughout the inferior temporal gyrus (Figure 5). We found a total 5,391 neurons in AD tissue (2.00% of all AD neurons) and 2,072 neurons in control tissue (0.49% of all control neurons) with enlarged neighbor distances greater than 60% (Figure 5D; *p* value = 0.0284), the cutoff derived from our experiments with rTg4510 mice. While their prevalence is related to AD status, 90.7% (±2.9%) of the neurons in AD tissue that have increased inter-neuronal distances did not contain an NFT, similar to the results in the tau transgenic mice.

Last, to better understand the processes by which cell death was occurring, we also investigated the size of neurons as changes in cell size may be indicative of specific cell death pathways. However, the sizes of neurons with enlarged nearest neighbor distances were not significantly different than neighboring neurons (*p* = 0.095). Collectively, these data suggest that increased inter-neuronal distances identify neurons at risk of dying in both tau transgenic mice and human AD, but not control, cortex.

## DISCUSSION

We have developed a method using advanced, longitudinal two-photon microscopy and quantitative volumetric image analysis to test the hypothesis that tangles precede neuronal death. We found that the opposite was true: the presence of a tangle reduces the risk of a neuron dying during the period of observation. Whether tangles act to sequester soluble, toxic forms of tau, reflect a successful upregulation of cytoprotective proteostatic mechanisms, or coincidently identify a neuronal subtype that is resilient, this result is contrary to expectations.

The current data show that neurons that overexpress mutant tau clearly die independently of developing a tangle. Neurons with NFTs also die but at a substantially lesser rate than non-tangle-bearing neurons in their vicinity. We hypothesize that the fibrilization of tau may be a marker for a resilience-related cell phenomenon that both predisposes toward aggregation of tau and, either due to this effect or to parallel processes, provides some protection. Recent analyses of transcriptomic data from an isolated AD tangle containing neurons determined that tangle-bearing neurons express multiple markers of cellular senescence.^14^ In senescence, apoptotic machinery is inhibited, and cells upregulate secretory proteins that typically have a negative paracrine effect on the surrounding tissue.^15^ Future exploration of the relationship between tangle formation and senescence will help us understand whether the improved survival of tangle-bearing neurons is desirable from a neural systems perspective.

Neuronal death is difficult to study in neurodegenerative diseases at any given moment since it is a rare, sudden event, and the cells of interest are defined by a phenotype—disappearance—that makes it impossible to observe them. These 4-dimensional high-resolution imaging studies allowed identification of a phenotype that appears to identify neurons at very high risk of undergoing neuronal death in the next several weeks. While it is possible that changes in non-neuronal cells (i.e., invasion of microglia and cytoskeletal reorganization in astrocytes) contribute to alterations in local microstructure, we favor the idea that the change in cortical microstructure may represent a form of cytotoxic edema prior to cell death.^16–18^ If so, agents targeting cytotoxic edema-related death mechanisms may prove of value in neurodegenerative diseases.

Using this morphological phenotype, we were able to identify “at risk” neurons in both mouse and human tissues, which rarely contained an NFT. This aligns with human neuropathological observations showing that the amount of neuronal death exceeds the number of tangles by 10 to 1, especially in the brain areas and lamina that develop both aggregates and neuronal loss.^4^ Similar observations have been made in frontotemporal dementia with tau pathology, Huntington’s disease, and dementia with Lewy bodies, where aggregates are not tightly linked to neuronal loss.^19–21^ Further, counting the number of ‘‘at risk’’ neurons in human in this study and assuming they die at a similar rate over the next several weeks as described in our longitudinal mouse experiments results in an estimated rate of neuronal loss of 5.5% (±1.5%) per year within the inferior temporal gyrus. This estimate agrees with previous reports from cross-sectional neuropathological studies and longitudinal magnetic resonance imaging that show changes in volume of the temporal lobe of ~3% per year.^4,22,23^

### Limitations of the study

Of note, rTg4510 mice overexpress by ~10-fold only a single isoform of tau (0N4R) containing the familial temporal dementia mutation P301L, which are factors that differ from human AD.^7,24^ Despite these differences, a recent proteomics analysis shows that the post-translational modifications impacting insoluble tau in these mice are reflective of those found in tissue from AD Braak I–III brains.^25^ Transgene insertion sites have also been mapped in this model and indicated a 244-kbp deletion in the fibroblast growth factor 14 locus (*Fgf14*),^26^ raising concern that some aspects of rTg4510’s phenotype is due to insertional effects. We propose that the cell death phenotype we observe in the rTg4510 cortex is due to tau overexpression, rather than transgene insertional effects, because of the following: (1) treatment with doxycycline, which restricts tau transgene expression, strongly suppresses the neuronal death phenotype, and (2) a completely independent tau transgenic model, ThyTau22, reveals an analogous phenotype. Moreover, careful evaluation of human cortex with AD showed similar alterations, clearly independent of any effects specific to mouse models. We conclude that tau is the likely driver of the cell death phenotype observed in both mouse lines.

There are other methodological details to consider that may also impact the interpretation of this work. For one, to track neurons, we used AAV to deliver the fluorescent nuclear tag under the *hSyn1* promoter. Prior studies have evaluated the tropism of these capsids for neurons and report that this is an effective system for delivering genes broadly to excitatory neurons^27–29^; however, this method may not have captured the full spectrum of disease-susceptible neurons. Similarly, two-photon imaging cannot penetrate the full thickness of the cortex, and deeper cortical neuron populations (or subcortical structures) are not well-represented in these data. Last, while our data in mice indicate that nearby neurons truly move farther away from dying neurons prior to their disappearance, similar longitudinal studies of individual cells cannot be performed in human tissues, and we consider the possibility that the increase in distances may also reflect the consequences of missing neurons. Future identification of additional molecular markers for this susceptible population could aid confirmation.

### Conclusion

In sum, this work provides evidence that non-tangle-bearing neurons are at higher risk for cell death than neurons containing fibrillar tau aggregates. Although not closely related to the presence of a tau fibrillar inclusion, the cell death process in the transgenic mice appears to be dependent on the presence of soluble tau. This observation challenges our assumption that NFTs cause neuron death, and instead, it indicates that alternative mechanisms for neurodegeneration in AD should be explored.

## STAR★METHODS

### RESOURCE AVAILABILITY

#### Lead contact

Further information and requests for resources and reagents should be directed to and will be fulfilled by the lead contact, Rachel E. Bennett (REBENNETT@mgh.harvard.edu).

#### Materials availability

This study did not generate unique reagents.

#### Data and code availability

- The datasets generated and analyzed during the current study have been deposited at Bioimage Archive and are publicly available. Accession numbers are listed in the key resources table.
- All original code has been deposited at Zenodo and is publicly available as of the date of publication. DOIs are listed in the key resources table.
- Any additional information required to reanalyze the data reported in this work is available from the lead contact upon request.

### EXPERIMENTAL MODEL AND STUDY PARTICIPANT DETAILS

#### Animals

A total of ten rTg4510 (Hemizygous for Tg(Camk2a-tTA)1Mmay, Hemizygous for Fgf14<Tg(tetO-MAPT*P301L)4510Kha>) and four control (Noncarrier, Hemizygous for Fgf14<Tg(tetO-MAPT*P301L)4510Kha>) mice were purchased from Jackson Laboratories (Strain #:024854; RRID:IMSR_JAX:024854). Cohort 1 consisted of *n* = 3 rTg4510 and *n* = 4 control mice that underwent cranial window imaging and histology ending at 6 months of age. Cohort 2 consisted of *n* = 3 rTg4510 mice that underwent cranial window imaging and doxycycline treatment and *n* = 4 rTg4510 littermates that were fed standard chow and reserved for protein biochemistry and histology ending at six months of age. Thytau22 mice are hemizygous for the transgene cassette expressing 4R MAPT with the G272V and P301S (RRID:MGI:3717255). Cohort 3 consisted of *n* = 3 Thytau22 and *n* = 4 wild-type littermates that underwent cranial window imaging and histology ending at 15 months of age. Cohort 4 consisted of *n* = 3 additional Thytau22 mice that were reserved for histology and protein biochemistry at 15 months of age. Both female and male mice were used in all experiments. Mice were housed under a 12-h light/dark cycle and provided food (Lab Diet cat no. RMH3000 5P75) and water *ad libitum*. For doxycycline treatment, mice were provided with a diet containing 200 mg/kg doxycycline (Bio-Serv cat no. S3888). All procedures involving mice were reviewed and approved by MassGeneral Brigham IACUC.

#### Human tissue samples

Autopsy tissue was collected at Massachusetts General Hospital, with informed consent of patients or their relatives. Fresh frozen human tissue was provided by the Massachusetts Alzheimer’s Disease Research Center (ADRC) with approval from the Mass General Brigham IRB (1999P009556). Six human participants with Alzheimer’s disease and five controls were selected from the ADRC. Sex, age at death, Braak staging, postmortem interval and comorbidities are listed in Table S3.

### METHOD DETAILS

#### Adeno-associated virus (AAV)

To generate a plasmid ligated the mKalama1-Nuc sequence into an existing AAV backbone containing the human synapsin1 promoter. pmKalama1-Nuc was a gift from Robert Campbell (Addgene plasmid # 14894; http://n2t.net/addgene:14894; RRID: Addgene_14894).^31^ The resulting plasmid was amplified using DH5α competent E. coli (ThermoFisher, # 12297016) and confirmed by restriction enzyme digest. AAV 2/8 was then produced by the Mass General Brigham Gene Transfer Vector Core with a final titer of 1.53E+13 GC/mL.

#### Surgical procedures

At 4 months of age, mice were anesthetized with ketamine-xylazine (100 mg/kg and 10 mg/kg respectively, administered intraperitoneally) and received a subcutaneous injection of 0.5% lidocaine in the scalp. After hair removal and swabbing with 7.5% povidone iodine and 70% ethanol, a scalp incision was made, and a 5 mm craniotomy was performed over the somatosensory cortex. After this, 1.5 μL of AAV was infused 0.3 mm below the surface of the brain using a 30 Ga Hamilton syringe and injector pump. Injections occurred at a rate of 0.2 μL/min and the needle was left in place for 5 min post-infusion. The brain was kept moist with PBS during this time. Following injection, an 8 mm cover glass was fixed in place using dental acrylic. Mice received buprenorphine (0.5 mg/kg subcutaneous) every 12 h and acetaminophen (300 mg/100mL in drinking water) for 3 days for analgesia and were closely monitored post-operatively. Following a three-week recovery period, mice were anesthetized with 5% isoflurane and received a retro-orbital intravenous injection of HS-84 (150 nmol, synthesized as described previously9) dissolved in PBS.

#### *In vivo* two photon imaging

For imaging, anesthesia was induced with 5% isoflurane and maintained with 2% isoflurane. Body temperature was regulated using a heating pad. A 70 kDA Texas Red conjugated dextran (12.5 mg/mL; Invitrogen, #D1864) was injected intravenously to label vasculature. Blood vessel labeling provided fiducial markers that assisted with locating regions of interest for imaging. Imaging was conducted using an Olympus FluoView FV1000MPE two photon laser-scanning system mounted on an Olympus Bx61WI microscope with an Olympus 25× 1.05 NA lens and an Insight X3-OL laser (Spectra-Physics). All images were collected using 800 nm excitation and three photomultiplier tubes (PMTs, Hamamatsu) that collected red, green and blue light corresponding to mKalama1 (peak em = 450 nm), HS-84 (peak em = 512,547 nm), and Texas red (peak em = 615 nm). For each ROI, z-stacks (XY 509 μm 3 509 μm; 1024 × 1024 pixels) were collected at 2-μm intervals to a ~200-μm depth from the cortical surface. Imaging parameters were reused between sessions.

#### Two photon image alignment and processing

Images were first processed in ImageJ to remove any fluorescence bleeding across channels. All weeks’ images from a single ROI were then loaded into Imaris (Bitplane; version 9.9.1) and aligned roughly from a top-down view of the XY plane by hand. Following rough alignment, the reference frame tool was used to place a reference frame for each image in the corner that can be used as an axis of rotation. The surface generation function was used, taking advantage of rolling-ball thresholding of signal, to generate surfaces around the mKalama1 stained neurons for each week of imaging. Each surface was assigned an ID as well as coordinates for its center point, which was then exported as an excel spreadsheet that could be read into a custom MATLAB (Mathworks, version r2022a) code ImageAlignment.mat that uses an existing function for solving absolute orientation.^32^ The ID of neurons stable across all weeks was used as fiducials for alignment and entered into ImageAlignment.mat.

Typically, 8 fiducials were chosen, 4 in each quadrant on the top of the sample, 4 in each quadrant on the bottom of each sample. The MATLAB code was then run, which identified the rotation, translation, and stretching of the frame necessary to align the fiducials that were entered, typically within 1–2 voxels. The image was cropped using the mask function to only show data that overlaps between all weeks of imaging. The Imaris Surface or Spots function was used to generate IDs and spatial statistics for the neurons or tangles that are present in each week of imaging. Finally, the neurons were manually counted to determine whether they disappear between weeks. Although we found it was possible to automate this analysis to some degree, manual verification was necessary to ensure neurons were disappearing and not a result of error.

#### Analysis of new tau tangle formation locations

A rolling ball thresholding (20 μm sphere) was used to isolate tau tangles in the HS-84 channel and form object surfaces in Imaris. Tangles were assigned with a minimum value cutoff of 100 voxels (49 μm^3^). New tangle formation was then verified by hand by comparing the location and shape of isolated HS-84 to data from the previous week, as well as identifying the presence of an associated neuron via mKalama1 staining.

#### Mouse histology

Mice were euthanized by CO_2_ asphyxiation and transcardial perfusion with PBS. Brains were removed and fixed in 4% paraformaldehyde for 48 h and then equilibrated in 30% sucrose in PBS. Forty-micron thick coronal sections were sliced on a freezing microtome. Sections were rinsed three times in Tris-buffered saline (TBS). For 3,3′-Diaminobenzidine tetrahydrochloride (DAB) staining only, sections were incubated for 10 min with 0.3% H_2_O_2_. Sections were then permeabilized and blocked for 30 min at room temperature with 0.25% Triton X- and 3% normal goat serum (NGS), respectively. Primary antibodies were diluted in fresh 3% NGS and 0.25% Triton X- solution and sections were incubated overnight at 4°C. The primary antibodies used are as follows: chicken anti-Green Fluorescent protein (GFP, 1:500, AvesLabs #GFP-1020; RRID: AB_10000240), mouse anti-phospho-tau (AT8, 1:500, Thermofisher #MN1020b; RRID:AB_223648) and rabbit recombinant anti-NeuN (1:500, Abcam #ab177487; RRID: AB_2532109). Anti-GFP was used to boost the signal intensity as it recognizes the epitope on mKalama1. Following three washes in TBS for 5 min each rinse, sections were incubated in secondary antibody diluted in 0.25% Triton X- TBS solution for 60 min at room temperature in a light-free environment. For The following secondaries were used: Alexa Fluor goat anti-chicken 647 (1:1000, Invitrogen) and Alexa Fluor goat anti-rabbit 555 (1:1000, Invitrogen). For DAB staining, biotinylated secondaries were used, followed by a 60 min incubation with streptavidin-HRP (Vector Laboratories, PK1000) and incubation with 0.25 mg/mL DAB containing NiCl_2_ and H_2_O_2_. After three additional rinses in TBS, sections were mounted on microscope slides in Immu-Mount (ThermoScientific) or Permount (Fisher Scientific) and coverslipped. Images were collected using an Olympus FV3000 confocal microscope or an Olympus VS120 automated slide scanner.

#### Human tissue histology

Histology was performed using a modified tissue clearing, staining, and imaging protocol described previously.^33^ Brain samples were thawed on ice then placed in 4% paraformaldehyde, made from diluting a 16% stock solution (Thermo Fisher Scientific, cat No. 50980487) in phosphate buffered saline (PBS), for 24 h at 4°C. Tissue was then rinsed with 50mL PBS five times over 24 h at 4°C. Tissue was then sliced on a Vibratome (Leica Biosystems, VT1000 S Vibrating Blade Microtome) to acquire 0.5mm thick sections of tissue.

Each tissue slice was incubated with 4% (w/v) acrylamide (Sigma-Aldrich, A3553) and 0.25% (w/v) VA-044 thermal polymerization initiator (Fisher Scientific, NC0632395) in PBS for 1 day at 4°C. It is important when preparing this solution to have the PBS pre-chilled and never warmed after adding the VA-044 until ready for the next step. After incubation, the tissue was placed in an X-CLARITY polymerization system (Logos Biosystems, South Korea) at 37°C for 3 h. Tissue was then rinsed with 50mL PBS for at least 3 times over 3 h to remove unreacted acrylamide.

Tissue slices were then placed individually into a 50mL falcon tube containing an aqueous delipidation solution composed of 200 mM sodium dodecyl sulfate (Sigma-Aldrich L3771) in 50 mM sodium borate (Sigma-Aldrich SX0355), brough to a final pH of 8.5–9 with sodium hydroxide (Sigma-Aldrich 795429). Tissue was then incubated on a shaker at 100 rpm and 37°C for 3 days. After delipidation the brain slices were rinsed with 50 mL PBS five times over 24 h, then the tissue was stored in PBS with 0.2% sodium azide or immediately used for immunohistochemistry.

Each brain slice was placed in a multi-well plate or Eppendorf tube that could hold the slice in an orientation allowing its large, flat sides to be exposed to solution. PBST (PBS with 0.2% Triton X-100, Thermo Fisher Scientific) was added to cover the top of the samples. Tissue was then heated to 50°C for 1 h then cooled down to room temperature prior to adding antibodies. The following conjugated antibodies were then added to the solution containing each tissue slice: phospho-tau (AT8, 1.6:500, Thermo Fisher Scientific Cat# MN1020, RRID:AB_223647) conjugated to Alexa Fluor 647 (Thermo Fisher, cat No. A37573), HuD Antibody E–1 (1.6:500, Santa Cruz Biotechnology Cat# sc-28299, RRID:AB_627765) conjugated to Alexa Fluor 555 (Thermo Fisher, cat No. A37571). Each slice was incubated individually with primary antibodies for one week at 4°C with gentle shaking. Following incubation, tissue was placed on shaker for one week at 4°C and rinsed several times with PBST to remove unbound and nonspecifically bound antibodies.

Samples were then incubated with 80% glycerol in water (v/v) for 24 h at room temperature with gentle shaking, then mounted on a glass microscope slide with a 0.5mm thick 3D-printed ring (Formlabs) to allow the tissue to remain in a pool of 80% glycerol during imaging. Tissue was imaged using an Olympus Inverted Confocal FV3000 with a 10× air objective. Multiple z-stacks were collected as tiles, then were stitched together using microscope software. Z-stacks were then reconstructed and visualized using either Imaris or ImageJ software.

#### Human image segmentation and quantification

Images were loaded into Imaris and fluorescence intensity in each plane was normalized using Imaris’ image-processing toolkit. Data were then exported as open-microscopy environment tiffs, loaded into ImageJ, split into individual channels, and converted to HDF5 format using Ilastik’s ImageJ plugin. The staining was then segmented using Ilastik’s pixel classifier workflow.^30,33^ The pixel classifier involved manual training using a paintbrush to draw over signal and background to help train the classifier on how to segment images. Images were then processed using the trained pixel classifier, and probability maps were exported as HDF5 format. Raw data and probability maps were then loaded into Ilastik’s object classification workflow, and manual assignment of objects as noise, cells, or other objects were used to manually train the object classifiers. Data was then processed as 500 × 500 × 20 blocks and applied over the entire image volumes. Data was then exported as object identities and spreadsheets with information about the objects’ classification and characteristics, such as their center-point location, which was then loaded into MATLAB code to determine nearest neighbor distances for every neuron identified in the tissue.

#### Protein biochemistry

To measure soluble and insoluble tau we performed sequential protein extraction using buffer containing 20 mM Tris-HCl, 0.15 M NaCl, 1 mM EGTA, 1 mM DTT, protease, and phosphatase-inhibitors with 1% Triton X- and then 1% sarkosyl. Protein extraction is described in greater detail in previously published protocols.^34,35^ The sarkosyl soluble and insoluble protein fractions were measured by BCA to determine protein concentration and 10 μg of each sample was loaded on a NuPAGE 4–12% Bis-Tris gel in MES SDS Running Buffer (Invitrogen). After running at 120V for 90 min, proteins were transferred to 0.2 μM nitrocellulose, rinsed in distilled water and incubated with Revert 700 total protein stain (LI-COR) to measure total proteins for normalization. Blots were then rinsed in TBS, blocked in Odyssey Blocking Buffer (LI-COR) and probed with mouse anti-human tau (HT7, Invitrogen, cat no. MN1000, RRID: AB_2314654) to measure total tau.

### QUANTIFICATION AND STATISTICAL ANALYSIS

#### Statistical analysis

The statistical test used for each experiment is indicated in the figure legends. Graphpad PRISM (version 9.5.0) was used to calculate the statistical significance for each test. Survival curves were calculated using Log rank Mantel-Cox test (Chi square = 92.65, df = 2, *p* value < .0001), log -rank test for trend (Chi square = 92.28, df = 1, *p* value < .0001), and Gehan-Breslow-Wilcoxon test (Chi square = 92.88, df = 2, *p* value < .0001). Analysis of variance (ANOVA) was performed using ordinary one-way ANOVA with Tukey’s multiple comparison test applied post hoc. Comparisons of neuron loss across multiple weeks was made using mixed effects analysis rather than repeated measures ANOVA due to the absence of 1–2 values for individual mice at late timepoints (due to cranial window changes over time). Hazard ratio was calculated by dividing the dying neurons by the total neurons for each group, then comparing the ratio of their survival rates. Chi-square value was calculated using the chi-square function and odds ratios were calculated by dividing ratios of values presented in the tables. Enlargement of nearest neighbor distances, including comparison of the population size with >60% values in human AD vs. control cases, was quantified using two-tailed t-tests. In all cases P-value thresholds were viewed as significant if < .05 and were represented in graphs with asterisks as follows as <.05 = *, <.01 = **, <.001 = ***, or ns if not significant.

## Supplementary Material

1

## Figures and Tables

**KEY RESOURCES TABLE:** 

REAGENT or RESOURCE	SOURCE	IDENTIFIER

**Antibodies**		

anti-Green Fluorescent Protein	AvesLabs	Cat#GFP-120; RRID: AB_10000240
mouse anti-phospho-tau, AT8	Thermo Fisher	Cat#MN1020b; RRID:AB_223648
rabbit recombinant anti-NeuN	Abcam	Cat#ab177487; RRID: AB_2532109
HuD Antibody E–1	Santa Cruz Biotechnology	Cat#sc-28299; RRID: AB_627765
mouse anti-human tau, HT7	Invitrogen	Cat#MN1000; RRID: AB_2314654

**Bacterial and Virus Strains**		

pmKalama1-Nuc	Robert Campbell	Addgene plasmid # 14894; RRID: Addgene_14894
DH5α competent E. coli	Thermo Fisher	Cat#12297016

**Biological Samples**		

Postmortem Human Tissue	Massachusetts Alzheimer’s Disease Research Center	N/A

**Chemicals, Peptides, and Recombinant Proteins**		

Doxycycline diet	Bio-Serv	Cat#S3888
Standard diet	Lab Diet	Cat#RMH3000 5P75
Texas Red conjugated dextran	Invitrogen	Cat#D1864
3,3’-Diaminobenzidine tetrahydrochloride	Thermo Fisher	Cat#J62216.09
Triton X-	Thermo Fisher	Cat#A16046.AP
normal goat serum	Thermo Fisher	Cat#50197Z
streptavidin-HRP	Vector Laboratories	Cat#PK1000
Immu-mount	Thermo Scientific	Cat#9990402
Permount	Fisher Scientific	Cat#SP15
Paraformaldehyde	Thermo Fisher	Cat#50980487
Acrylamide	Sigma-Aldrich	Cat#A3553
VA-044	Fisher Scientific	Cat#NC0632395
Sodium dodecyl sulfate	Sigma-Aldrich	Cat#L3771
Sodium borate	Sigma-Aldrich	Cat#SX0355
Sodium Hydroxide	Sigma-Aldrich	795429
Alexa Fluor 647	Thermo Fisher	Cat#A37573
Alexa Fluor 555	Thermo Fisher	Cat#A37571
EGTA	Thermo Fisher	Cat#E1219
DTT	Thermo Fisher	Cat#R0861
Protease and Phosphatase inhibitor cocktail	Thermo Fisher	Cat#78446
Sarkosyl	Sigma-Aldrich	Cat#L5777
Revert 700 total protein stain	LI-COR	Cat#926–11010
NuPage 4–12% Bis-Tris gel	Thermo Fisher	Cat#NP0322BOX
HS-84	Peter R. Nilsson	Synthesized following R.A. Simon et al. (2014), 10.1002/chem.201402890

**Deposited Data**		

Mouse longitudinal imaging data	BioImage Archive (https://www.ebi.ac.uk/bioimage-archive/)	S-BIAD1251
Human tissue images	BioImage Archive (https://www.ebi.ac.uk/bioimage-archive/)	S-BIAD1251

**Experimental Models: Organisms/Strains**		

rTg4510 mice (Hemizygous for Tg(Camk2a-tTA)1Mmay, Hemizygous for Fgf14<Tg(tetO-MAPT*P301 L)4510Kha>)	Jackson Laboratories	Strain #:024854; RRID:IMSR_JAX:024854
rTg4510-control littermate mice (Noncarrier, Hemizygous for Fgf14<Tg (tetO-MAPT*P301L)4510Kha>)	Jackson Laboratories	Strain #:024854; RRID:IMSR_JAX:024854
ThyTau22 mice (hemizygous for the transgene cassette expressing 4R *MAPT* with the G272V and P301S)	Luc Buee	RRID:MGI:3717255

**Software and Algorithms**		

Imaris	Oxford Instruments	N/A
Ilastik	*ilastik: interactive machine learning for (bio)image analysis* ^30^	N/A
Graphpad Prism (9.5.0)	Graphpad	N/A
ImageAlignment.mat	This paper	https://doi.org/10.5281/zenodo.12754178
PolygonNeuronDisappearance.mat	This paper	https://doi.org/10.5281/zenodo.12754178
AnalyzeNN.mat	This paper	https://doi.org/10.5281/zenodo.12754178
Multipage Tiff stack	MATLAB Central File Exchange	Yoon-Oh Tak (2024). Multipage TIFF stack (https://www.mathworks.com/matlabcentral/fileexchange/35684-multipage-tiff-stack)
Absolute Orientation - Horn’s Method	MATLAB Central File Exchange	Matt J (2024). Absolute Orientation - Horn’s method (https://www.mathworks.com/matlabcentral/fileexchange/26186-absolute-orientation-horn-s-method)

## Highlights

- Two-photon microscopy methods track tau tangle formation and neuron loss in live mice
- In mouse models, neurons without tangles die more frequently than neurons with tangles
- Dying neurons can be identified by changes in nearby cortical microstructure
- Human Alzheimer’s brain shows the same microstructural alterations observed in mice
