# Supplementary material for: Neurofibrillary tangle-bearing neurons have reduced risk of cell death in mice with Alzheimer’s pathology
Source: Cell Rep. Author manuscript; Available in PMC 2024 Sep 30. (PMC11441076; doi:10.1016/j.celrep.2024.114574)
Supplement: 1 [file NIHMS2019531-supplement-1.pdf]

Cell Reports, Volume *43*

Supplemental information

Neurofibrillary tangle-bearing neurons have reduced risk of cell death in mice with Alzheimer's pathology

Theodore J. Zwang, Eric del Sastre, Nina Wolf, Nancy Ruiz-Uribe, Benjamin Woost, Zachary Hoglund, Zhanyun Fan, Joshua Bailey, Lois Nfor, Luc Buée, K. Peter R. Nilsson, Bradley T. Hyman, and Rachel E. Bennett

Supplemental Information

Title: Neurofibrillary Tangle-Bearing Neurons have Reduced Risk of Cell Death in Mice with Alzheimer's Pathology

Authors: Theodore Zwang[1, 2], Eric del Sastre, Nina Wolf, Nancy Ruiz-Uribe, Benjamin Woost, Zachary Hoglund, Zhanyun Fan, Joshua Bailey, Lois Nfor, Luc Buée, K. Peter R. Nilsson[3], Bradley T. Hyman[1,2,ŧ,*], Rachel E. Bennett[1,2, ŧ,*]

Contents: 2 figures, 3 tables

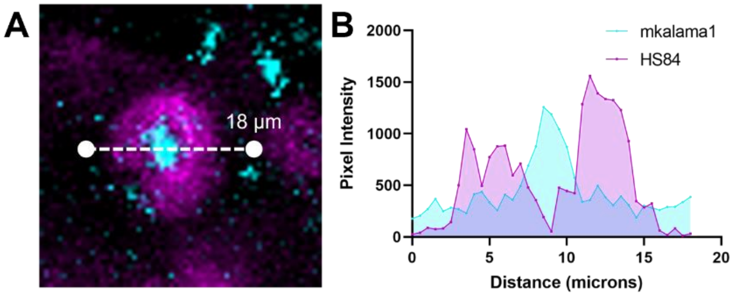

Supplemental Figure 1. Example of a typical tangle-bearing neuron imaged by multiphoton microscopy. (**A**) Neurons expressing nuclear mKalama1 (cyan) and that bound HS-84 (magenta) were readily detected in the brains of rTg4510 mice. (**B**) The fluorescence intensity along a line drawn across the neuron in panel A is plotted, showing mKalama1 nuclear labeling and HS-84-positive somatic tau aggregates in this cross-sectional image.

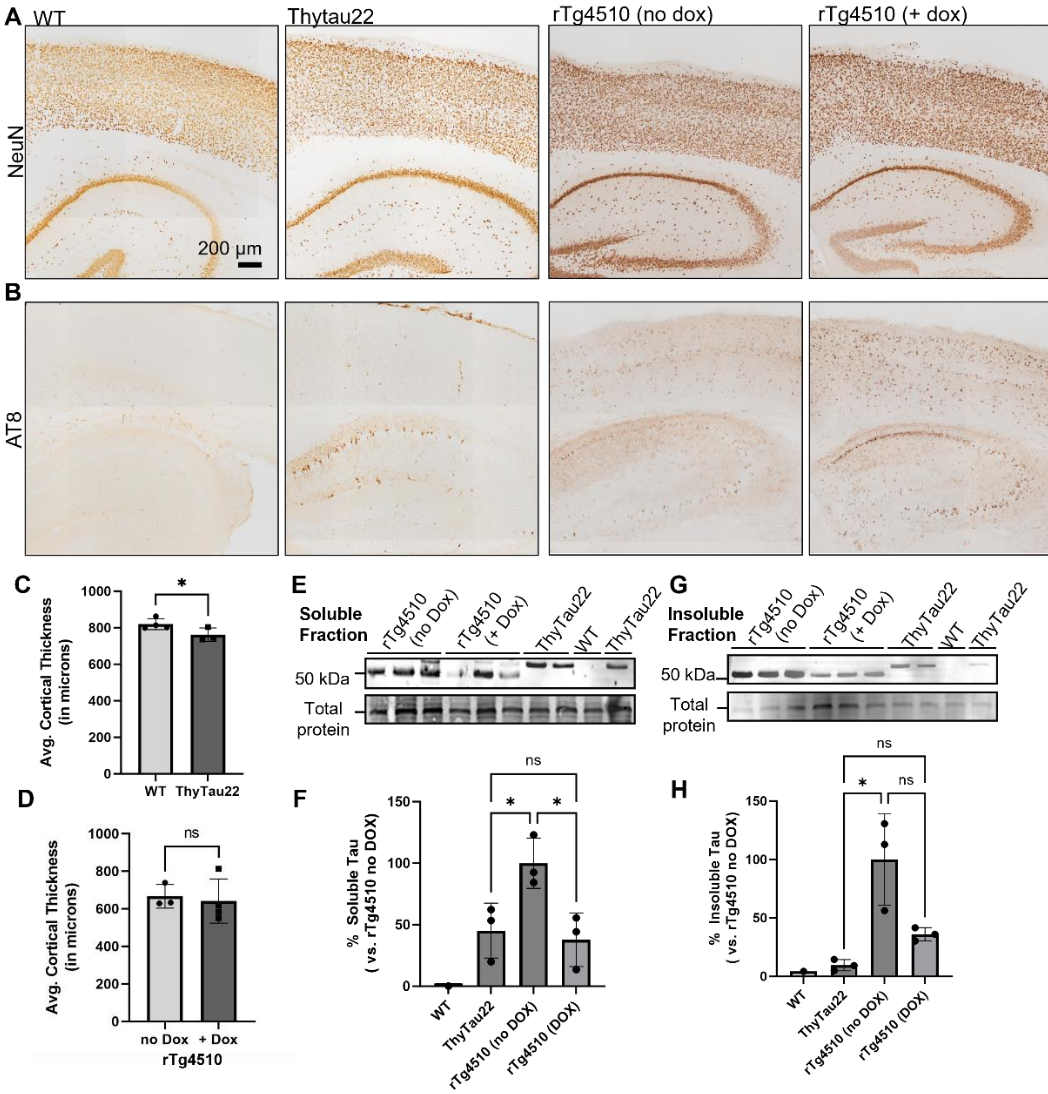

Supplemetal Figure 2. Cortical atrophy and tau in mouse models. (**A**) Histology showing NeuN-labeled neuronal nuclei in wild-type (WT) and Thytau22 mice as well as in rTg4510 mice that did not receive doxycycline treatment (no dox) and that did (+ dox). (**B**) Representative histology from WT and Thytau22 mice showing tau tangles (AT8, ptau202/205). (**C**) Quantification of cortical thickness from NeuN labeled sections comparing Thytau22 and wild-type littermate controls (t-test, * = p<0.05). (**D**) Quantification of cortical thickness from NeuN labeled sections comparing rTg4510 mice with or without 1 month of doxycycline treatment (t-test, p>0.05). (**E**) Western blot showing HT7 (total human tau antibody) labeled sarkosyl soluble protein fraction and total protein stain used for normalization. (**F**) Quantification of soluble tau from western blot (ANOVA, * = p<0.05). (**G**) Western blot showing HT7 (total human tau antibody) labeled sarkosyl insoluble protein fraction and total protein stain used for normalization (**H**) Quantification of insoluble tau from western blot (ANOVA, * = p<0.05).

	Neurons w/ NFT	*Neurons w/o NFT*
Persist	229	1314
Die	6	172

Supplemental Table 1 Chi-square table showing the number of neurons with and without neurofibrillary tangles and whether they persist or die over the course of imaging. Chi-square test shows a statistically significant difference (Chi-square = 17.8, df = 1, $p<0.0001$) with NFT-bearing neurons being 78% less likely to die overall (odds ratio).

	Dying Neurons	*Persistent Neurons*
Swelling	20	23
Not Swelling	24	90

Supplemental Table 2. Chi-square table showing the number of neurons in rTg4510 mice that are dying and persistent across weeks and whether the space around them shows swelling.. Swelling neurons are defined as an increase in volume change (Figure 4B,D) across consecutive weeks that is greater than 4 SE above the median value for the entire population (>21.3% increase in volume). Chi-square test shows a statistically significant difference (Chi-square = 10.03, df = 1, p = 0.0015) with swelling neurons having an increased risk of death of 121% (odds ratio).

ADRC Donor	Sex	Age at Death	Braak Stage	Comorbidities	PMI	ADNC A	ADNC B	ADNC C	Disease Duration
AD1	F	≥90	VI	Arteriolosclerosis	5	3	3	3	Not reported
AD2	F	70	VI	CVD	24	3	3	3	13 Years
AD3	M	≥90	III	CVD	14	3	2	2	3 Years
AD4	M	75	VI	CVD	4	3	3	3	8 Years
AD5	F	66	VI	CVD	14	3	3	3	10 Years
AD6	M	74	V	CVD	10	3	3	3	5 Years
CON1	F	56	0	Acute Hypoxia	8	1	0	0	-
CON2	F	64	I	CVD	18	0	N/A	N/A	-
CON3	M	62	0	CVD	17	1	0	0	-
CON4	M	73	0	CVD	14	1	0	1	-
CON5	F	68	I	CVD	20	0	1	0	-

Supplemental Table 3. Demographics and characteristics of all donors used in this study including sex (M = Male ; F = Female), Age at death, Braak stage, Comorbidities (CVD = Cerebrovascular Disease), Post Mortem Interval (PMI; hours). CON = normal aging control.
